# COVID-19 in children: considerations for returning to school^☆^

**DOI:** 10.1016/j.bjorl.2020.09.005

**Published:** 2020-10-06

**Authors:** Alexandre Caixeta Guimarães, Luciana Becker Mau, Rebecca Christina Kathleen Maunsell

**Affiliations:** a Universidade Estadual de Campinas (UNICAMP), Campinas, SP, Brazil; b Instituto de Infectologia Emilio Ribas, São Paulo, SP, Brazil

The year 2020 has been marked by the pandemic caused by the SARS-COV2 virus, which started in China at the end of 2019 and spread to all continents within a few months. In Brazil, cases began to emerge in March and in an attempt to reduce the increase in the number of cases, several measures were adopted by the authorities at the municipal, state and federal levels, aiming to flatten the case curve.

Among these measures we can mention social isolation with different levels of quarantine, campaigns to encourage people to stay at home, incentive to use facial masks, frequent handwashing, use of gel alcohol and the closing of schools and universities.

Brazil as a whole has reached a plateau stage regarding the number of cases, but because it is a continental country, the reality is quite distinct in different regions. In many places, the pandemic is already decelerating regarding the daily number of deaths. With the change in the epidemiological scenario, measures are gradually being taken to make social isolation more flexible and allow the safe return to normal activities, while following safety protocols.

A topic that has been debated is when in-person classes for students should be resumed at schools. Some points must be emphasized for us to understand what the risks are in resuming in-person classes.

The incidence of diagnosed coronavirus cases in children under 18 years of age is approximately 2% of the total cases;^1^ however, if we consider that children represent 20% of the total population, one can infer that the chance of a child becoming infected is about 10-fold lower than that of the rest of the population. Several hypotheses have been developed to explain the lower incidence of COVID-19 in children, such as a more active innate immune system, less exposure to cigarettes and pollutants, and immaturity of angiotensin-converting enzyme 2 (ACE-2) receptors, which are binding sites for the coronavirus to enter the cells.^1^

Up to 90% of COVID-19 cases in children are asymptomatic or mild, and the most frequent clinical manifestations are cough (49%), fever (47%), odynophagia (36%) followed by diarrhea or vomiting (17%) and rhinorrhea (9%).^1^ In adolescents, dizziness, myalgia and chills are relatively more common. Severe cases requiring hospitalization are more common in children under 2 years of age, followed by preschoolers and schoolchildren, with severe cases being rarer in adolescents. Cases requiring ICU admission are rare and mortality reported to be 0.03% in a group of children aged 5–17 years.^2^

In contrast to what occurs with the influenza virus, children's role in the transmission of SARS-COV-2 seems to be small. Data suggest that, in most cases, children acquire the virus from an adult at home and there have been rare cases of known transmission from one child to another.^3^ In a study that assessed transmissibility from 18 cases of infected children in Australia, transmission occurred at school in only 2 cases, in one case by another adolescent and in the other by an infected teacher.^3^ In Sweden, no increase in the number of children infected during the pandemic was observed, despite the fact that schools remained open.^3^ On the other hand, there was an outbreak of COVID-19 cases 10 days after schools reopened in Israel, in a scenario where mitigation measures such as wearing facial masks were not followed due to exceptional hot weather situations, which led to the closing of schools. Considering the low incidence of COVID-19 cases in children, the high proportion of asymptomatic cases and the low known transmissibility among children, the closing of schools seems to have little impact as a pandemic control measure.^4^

We cannot ignore the negative consequences that the closing of schools impose on children; the level of physical inactivity has increased, children in particular are practicing less physical activity and spending more screen time due to the use of cell phones, television or the computer. Social interaction with other children has been reduced and the sudden change in routine in children's lives is increasing the number of psychiatric disorders, such as anxiety, depression, post-traumatic stress disorder, sleep disorders and behavioral changes.^5^ The closing of schools can also cause problems for childrens’ nutrition, as in many cases, the childrens, main meals and sources of calories occur in the school environment.

To date, schools have been closed for more than 200 days in Brazil; in most of the other developed countries, such as Denmark, France and Germany, schools have been closed for a much shorter period. It is possible that the closing of schools for a longer period may cause greater damage to society than their reopening with some care measures. Children with uncontrolled pulmonary or cardiovascular comorbidities are at increased risk for developing severe forms of COVID-19 and their return to school should occur at a later date. Likewise, the return of those who have contact with higher risk groups at home should also be postponed. It is also important to postpone face-to-face activities for teachers and employees who belong to or live with groups at higher risk. The contamination between adults who interact in the school environment is not higher than that observed at home or in the community. Regarding the contamination of adults by children that returned to school: in studies with children who tested positive for COVID-19, where there was follow-up of individuals who had contact with the children, no positivity was observed in adults who had contact with them in the school environment.

There is limited evidence that school has a relevant role in COVID-19 transmission in the community; however, there are indications that community transmission can be imported into and reflected in the school setting. Apparently, the control of community mitigation measures such as social distancing, cancellation of mass gatherings, hand hygiene and isolation in case of symptoms are of utmost importance and would be enough so that the return to the school environment does not represent a greater risk than exposure to other environments.

What would be our role as doctors in this scenario? We believe we must inform our patients of the real situation and health risks that children and adolescents will face with the reopening of schools, consider the losses caused by the lack of school for them and we must also remember to recommend the care measures that must be taken at the time of returning to school, mainly emphasizing that when they have symptoms, even if mild ones, of a possible coronavirus condition, they should stay away from the school until the condition is resolved. The return to in-person activities must be gradual, optional and careful for both students and parents, teachers and other professionals.

## Conflicts of interest

The authors declare no conflicts of interest.

## References

[1] Zare-Zardini H., Soltaninejad H., Ferdosian F., Hamidieh A.A., Memarpoor-Yazdi M. (2020). Coronavirus disease 2019 (COVID-19) in children: prevalence, diagnosis, clinical symptoms, and treatment. Int J Gen Med..

[2] Wiersinga W.J., Rhodes A., Cheng A.C., Peacock S.J., Prescott H.C. (2020). Pathophysiology, transmission, diagnosis, and treatment of coronavirus disease 2019 (COVID-19): a review. JAMA..

[3] Williams P.C.M., Howard-Jones A.R., Hsu P., Palasanthiran P., Gray P.E., McMullan B.J. (2020). SARS-CoV-2 in children: spectrum of disease, transmission and immunopathological underpinnings. Pathology..

[4] Davies N.G., Klepac P., Liu Y., Prem K., Jit M., CMMID COVID-19 working group, Eggo R.M. (2020). Age-dependent effects in the transmission and control of COVID-19 epidemics. Nat Med..

[5] Marques de Miranda D., da Silva Athanasio B., Sena Oliveira A.C., Simoes-E-Silva A.C. (2020). How is COVID-19 pandemic impacting mental health of children and adolescents?. Int J Disaster Risk Reduct..

